# Local Neutral Networks Help Maintain Inaccurately Replicating Ribozymes

**DOI:** 10.1371/journal.pone.0109987

**Published:** 2014-10-09

**Authors:** András Szilágyi, Ádám Kun, Eörs Szathmáry

**Affiliations:** 1 Parmenides Center for the Conceptual Foundations of Science, Munich/Pullach, Germany; 2 MTA-ELTE Theoretical Biology and Evolutionary Ecology Research Group, Budapest, Hungary; 3 Department of Plant Systematics, Ecology and Theoretical Biology, Institute of Biology, Eötvös Loránd University, Budapest, Hungary; 4 MTA-ELTE-MTM Ecology Research Group, Budapest, Hungary; Fred Hutchinson Cancer Research Center, United States of America

## Abstract

The error threshold of replication limits the selectively maintainable genome size against recurrent deleterious mutations for most fitness landscapes. In the context of RNA replication a distinction between the genotypic and the phenotypic error threshold has been made; where the latter concerns the maintenance of secondary structure rather than sequence. RNA secondary structure is treated as a proxy for function. The phenotypic error threshold allows higher per digit mutation rates than its genotypic counterpart, and is known to increase with the frequency of neutral mutations in sequence space. Here we show that the degree of neutrality, i.e. the frequency of nearest-neighbour (one-step) neutral mutants is a remarkably accurate proxy for the overall frequency of such mutants in an experimentally verifiable formula for the phenotypic error threshold; this we achieve by the full numerical solution for the concentration of all sequences in mutation-selection balance up to length 16. We reinforce our previous result that currently known ribozymes could be selectively maintained by the accuracy known from the best available polymerase ribozymes. Furthermore, we show that *in silico* stabilizing selection can increase the mutational robustness of ribozymes due to the fact that they were produced by artificial directional selection in the first place. Our finding offers a better understanding of the error threshold and provides further insight into the plausibility of an ancient RNA world.

## Introduction

Ever since the insight of Manfred Eigen [1], researchers have been puzzled by the question how the adverse effect of high mutation rate on the selectively maintainable genome size could be alleviated. The classical, sequence-based error threshold looks like this: imagine a population of wild-type (also called master in this context) and mutant templates of uniform length replicating with a finite accuracy. We further assume that wild-type sequences have high fitness and all the mutant copies have (identical) low fitness. This is obviously a simple fitness landscape. Whereas Eigen's [1] formalism can handle arbitrary fitness landscapes, the derivation of the error threshold is much more straightforward for this simple case. If we further adopt the simplification of no back mutations then a very simple result follows [2] for the critical error rate, 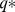:
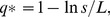
(1)


where 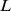 is the length of the sequence and *s* is the selective superiority of the wild-type sequence. An error rate of 1%, which is already quite an optimistic assumption, allows a sequence not longer than 100 nucleotides to be maintained. Four decades ago this problem looked rather paralyzing: what could a peptide enzymatically do that consisted of a mere 33 amino acids? And even if short peptides could be sufficiently enzymatic, does one gene make a genome?

In an RNA world [3]–[8], in which RNAs act both as information storage molecules and enzymes, things are likely to have been different. There are ample examples of ribozymes that are less than a 100 nucleotides long [4], [9] (see also Table S1). Actually, the smallest ribozyme is 5 nucleotides long [10]. On the other hand, while a ribozyme can be less than 100 nucleotides long, a single gene still does not make a genome. However, recent investigations have somewhat alleviated the error threshold problem. First, it seems that intragenomic recombination may have shifted the threshold by about 30% [11]. Second, the processivity of replication (i.e. the constraint that during enzymatic template replication nucleotides have to be inserted one by one into the growing copy, and this must happen repeatedly) could have worked against erroneous insertions that slowed down replication: erroneous copies would have thus suffered from a built-in fitness disadvantage [12]. Although this effect was shown to be considerably smaller for RNA than DNA, nevertheless it may also have alleviated the error threshold by about one-third. Third, as we have shown by the analysis of two existing ribozymes (the *Neurospora* VS [13] and the hairpin ribozyme [14]), the fact that the maintenance of structure is more important for function than that of sequence significantly shifts the error threshold to longer sequences (the genotypic and phenotypic error thresholds are 0.033 versus 0.053 and 0.042 versus 0.144 for the two ribozymes, respectively), in support of the investigations of Takeuchi *et al.* [15] and Reidys *et al.* [16]. They proposed that neutral mutations, by keeping the same phenotype, should modify the error threshold:
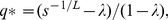
(2)


where the critical parameter 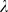 is the degree of neutrality, i.e. the fraction of neutral mutants among the mutants one step away from the master phenotype (the formula is from [15]).

This phenotypic error threshold suggested two important considerations: (1) known ribozymes by the virtue of their small sizes could be replicated by replicases whose accuracy would not have surpassed those of experimentally produced, available polymerase ribozymes (working with error rates in the range 0.04–0.01 per digit per replication [17], [18]), and (2) a replicase working at an error rate one magnitude lower than the currently known polymerase ribozymes could have replicated a small genome of a complete ribo-organims [19], [20].

In this paper, we broaden the investigation of the error threshold into important directions. The questions are:

(1) What structural characteristic of RNAs determines the position of the phenotypic error threshold? More specifically, can the degree of neutrality (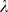) be employed to estimate the error threshold as proposed in [15], [16]. Please note, that the formula in Eq. 2 was derived by assuming that the effect of mutations are independent, and thus if there is two mutations that are independently neutral, then a sequence having both of them together will still be neutral. This is not necessary true. Furthermore the degree of neutrality is assumed to be the same for every sequences of the master type. We know that there places of different degree of neutrality along neutral paths (series of sequences having the same phenotype) [21]. Moreover, note that this formula is obtained at zero concentration of the master phenotype, which condition cannot occur when there is back mutation, especially in case of short sequences; it therefore gives an overestimate of the error threshold. In our analysis we start from Eigen's quasispecies model [1] and based on fitness landscapes of folded RNA we analytically calculate the error threshold, and correlate it with structural characteristic, thereby checking Eq. 2.

(2) How general is our previous finding [19] that even low-accuracy replicases could replicate the known ribozymes if only the former were processive enough (i.e. if they could replicate adequately long templates irrespective of the accuracy problem)? Note that the best experimentally verified polymerase ribozyme, while being 198 nt long, can copy sequences up to 95 nt [17] or can copy a very specific template up to 206 nt [22]. If Eq. 2 can be used to estimate the error threshold, then we can make a rough estimate for known ribozyme sequences from the literature, and strengthen (or disprove) our previous claim.

We consider the above raised questions in turn. Finally, we look at the world of putative ribo-organisms in the light of our findings.

## Results

The position of the error threshold for an arbitrary fitness landscape and in the presence of back mutations is a matter of definition in the quasispecies model of Eigen [1], [23], [24]. We have calculated the error threshold for binary (GC) sequences (and the phenotypic error threshold for associated secondary structures) up to length 16. Sequences comprising of only GC nucleotides have similar structural diversity as those composed of all four bases (see below), and thus our results are representative for them as well. Note that even at this length, sequence space is vast (there are 2^16^ = 65536 possible sequences) and exhaustive calculations for longer sequences or sequences with four bases are technically not feasible at the moment. Since the sequences are relatively short in this exhaustive analysis, the error threshold is not as sharp as for longer ones [25], and other types of diagnostics (such as the avoided crossing of the first and second largest eigenvalues [26]) do not work either, we define the error threshold as the error rate where the total concentration of master templates equals that of the non-master.

We employ a simple fitness landscape in which sequences belonging to the same secondary structure class (SSC) defined as the set of sequences of identical length sharing the same secondary structure, have high fitness (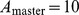) and all other sequences have base fitness (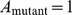). The selective superiority is thus 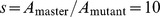. The minimum free energy structures of the sequences are obtained with the ViennaRNA Package ver. 1.8 [27]. For a given SSC, we set the so called value matrix (cf. Eq. 6) of the system (cf. Eq. 5), which contains the replication and degradation rate constants of the sequences, according to the secondary structure corresponding to the SSC. By computing the leading eigenvector at a given per digit replication accuracy *q*, we get the equilibrium densities of master and mutant sequences. The value of *q* at which the densities of master and mutant sequences equal defines our error threshold. (Note that in case of *L = 16*, the value matrix has 2^32^≈4.3•10^9^ entries; memory consumption and computation time for longer sequences is enormous). The error threshold for major SSCs (SSCs covering at least 0.1% of the sequence space) is calculated.

**Figure 1:**
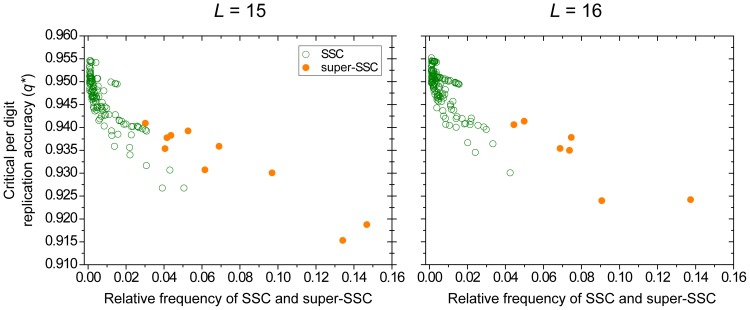
Error thresholds of secondary structure classes (SSCs). The graphs depict the critical per digit replication accuracies (error thresholds) as a function of the frequency of sequences belonging to an SSC among all possible structures of 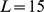 (left) and 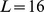 (right). Open circles represent individual SSCs, solid circles represent super-SSCs (SSSC) that merge structures that only differ in the flanking single-stranded regions. Only SSCs are included that cover at least 0.1% of the total sequence space.

**Figure 2:**
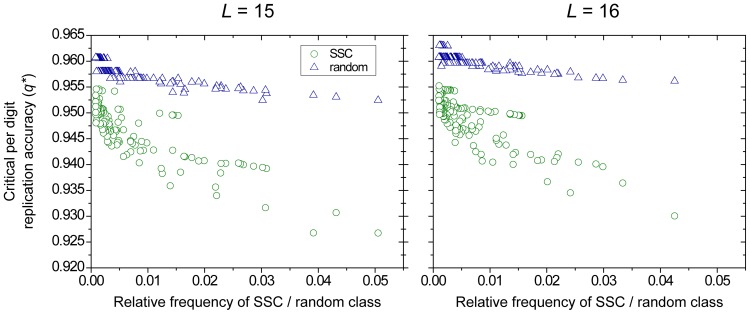
Error threshold for random sequences. The green circles represent SSCs based on secondary structures (as in Fig. 1), blue triangles represent the error threshold of classes of random sequences of the same size as the corresponding SSC. Results are for 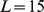 (left) and 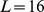 (right).

We find that the error threshold of sequences whose structures belong to the same SSC scales inversely with the relative frequency of the SSC genotypes in sequence space (Fig. 1): more common secondary structures are more robust. SSCs consisting of more sequences have a lower critical per digit replication accuracy, hence a more permissive error threshold. This can be understood as a higher number of members translate to a larger neutral network in the sequence space [28]. However, it is not just the mere number of sequences belonging to the class which makes them more robust against errors: Fig. 2 clearly shows that sets of random sequences, even if they have the same size as a SSC, suffer from a remarkably stricter error threshold.

A way to extend the investigation of robustness towards more frequent structures is to merge structurally similar SSCs forming a super-SSC. Super-SSC is defined as structures that only differ in the number of leading and trailing single stranded nucleotides (a complete list of super-SSC is found in Table S3).With super-SSCs, the number of sequences belonging to a class can be increased while the main feature of the secondary structure (i.e. the lengths of stem and loop but not their positions in the chain) still remains the same. The above finding still holds for super-SSCs (see Fig. 1, red dots), with super-SSCs having a higher error threshold than any of the error thresholds of their SSCs. Thus if only the major structural features are selected for, the error threshold is even more permissive.

**Figure 3:**
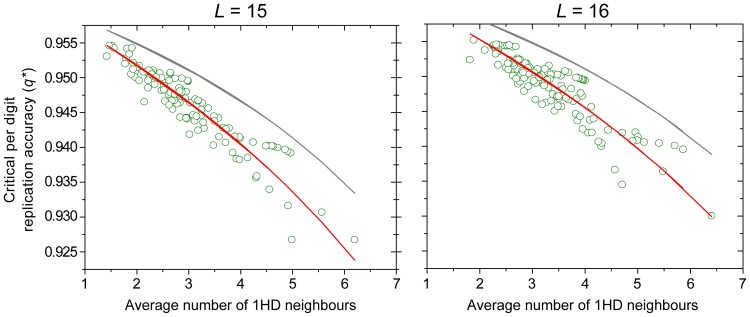
Correlation of the error threshold with average number of 1–Hamming distance neighbours. Critical per digit replication accuracy of SSCs as a function of the average number of 1–Hamming distance neighbours for sequences in the SSC. Red curves show fit of Eq. 3 to the data points, while the dark gray curve show fit to Eq. 3 with no back-mutations (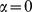). Results are for 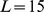 (left) and 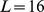 (right). The average number of 1HD neighbours can be transformed to 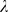 by dividing it by the length of the sequence.



Next, we show that the phenotypic error threshold can be estimated by calculating the fraction of neutral 1-mutant neighbours. (It was previously hinted that it might be sufficient to consider the neutral mutants being just one mutation step away from the master [29]). We have found that for short sequences, the error threshold scales *almost* linearly with the average number of 1-mutant neighbours in the SSC (Fig. 3), which supports the insight provided by the Takeuchi-Hogeweg formula (Eq. 2). If we introduce the simple assumption that the frequency of back mutations is proportional to the number of 1-step neutral mutants there is a strong correlation between empirical calculations and the corrected Takeuchi-Hogeweg formula for error threshold (cf. Eq. 18 in Methods and the Discussion):

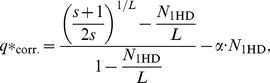
(3)where *s* is the selective superiority of the focal phenotype, *N*_1HD_ is the number of neutral 1-Hamming distance neighbours and *α* is the proportionality factor of back mutation. This correction includes the fifty-fifty definition of the error threshold given above and a heuristic account of the effect of back mutations.

We conclude thus that there is an ordering of robustness for two (or more) sequences of identical lengths: the one having more neighbours a single mutation step away with the same phenotype tends to have a higher error threshold.

In order to apply the formula (Eq. 2) to calculate the error threshold, we need the length *L* of the sequence, the frequency 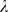 of one-step neutral mutants among all one-step mutants and the selective advantage *s* of the master phenotype. The length is naturally given, and 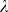 can be calculated exhaustively by folding all possible such mutants and comparing their minimum free energy structures to the secondary structure of the original sequence (this neglects mutations that are harmful even though the secondary structure remains unchanged) as 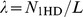 (there are 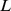 such mutant sequences for sequences comprising of only two bases, while there are 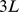 such sequences if all four bases are considered) (see Methods for the detailed explanation of the determination of 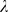). For the selective advantage, we apply our previous estimate of 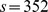 obtained for two fitness landscapes sampled more exhaustively [19].

**Figure 4:**
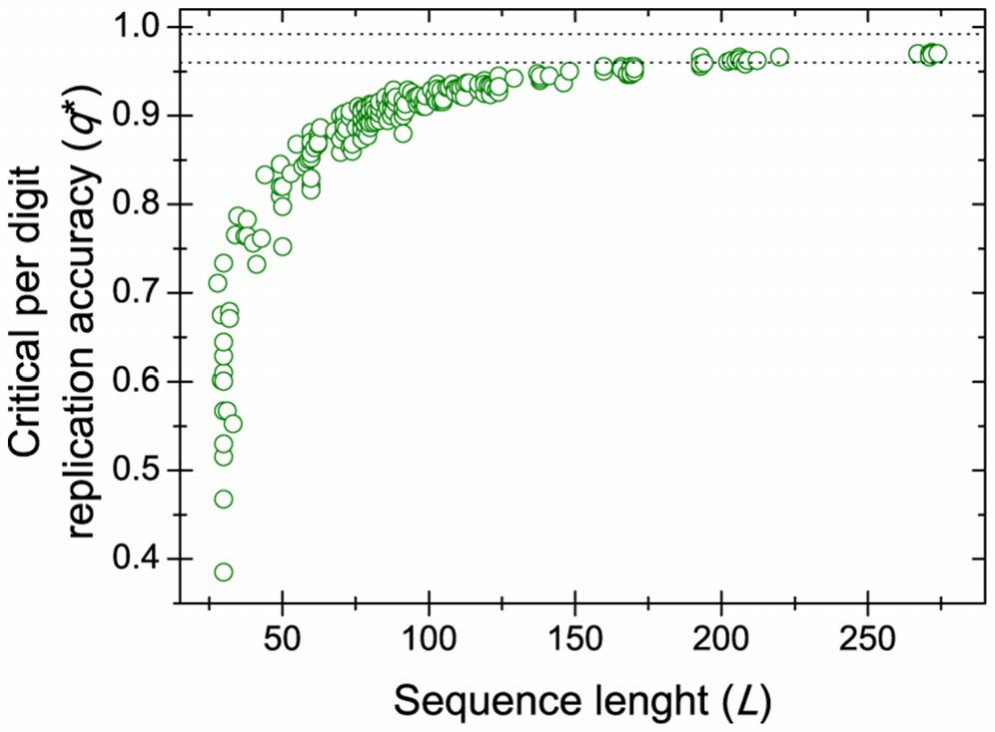
Error threshold of real ribozymes and aptamers. Critical per digit replication accuracy required to replicate real ribozymes and aptamers, calculates using Eq. (2). Each point represents a ribozyme or an aptamer (see Table S1). The two dotted lines mark the zone of replication accuracy of putative RNA-dependent RNA polymerases (0.96 <q <0.99).

Now we turn to the case of real ribozymes and aptamers from the Aptamer Database [30] and from the review of Chen and coworkers [9], providing 305 sequences altogether [31]–[113] (Table S1). This set of sequences represents a considerable fraction of all known aptamers and ribozymes whose functions have prebiotic significance. The ribozymes in particular were selected on the basis of their metabolic importance which suggests their prebiotic significance. It turns out that all of these ribozymes and aptamers have lower critical copying fidelity than the 99% fidelity of the most recent polymerase [17] and most have a critical copying fidelity lower than the average 96.5% fidelity reported for the first putative polymerase [18] (Fig. 4). Thus moderately sized, metabolically important ribozymes can be replicated despite rather low fidelities (high error rates).

**Figure 5:**
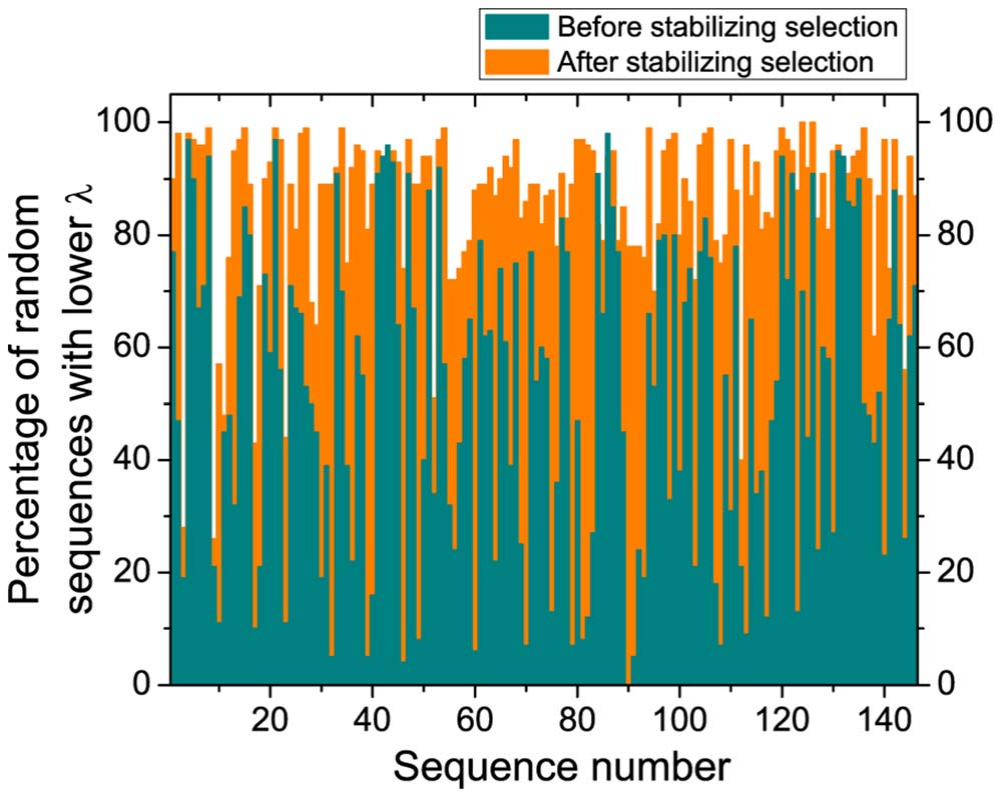
Stabilizing selection increases robustness of sequences. The percentage of the random sequences having lower fraction of 1-step neutral mutants among all 1-step mutants (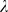) than the original sequence (green bars) and the best sequence after 5000 generations of stabilizing selection (orange bars). Sequences are ordered according to their length. Exact 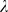 are given in Table S1.

Another appreciation of the calculated error thresholds is possible as follows. For every length of aptamers or ribozymes in Table S1, we have folded 1000 randomly chosen RNA sequences of the given length. Of course, this is a small sample, but with good chance we mainly obtain structures that are common in phenotype space [114]. Enzymes are likely to belong to one of these common structures [115], [116]. We have collected λ for all of the 1000 sequences which tells us how the degree of neutrality (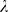) of aptamers/ribozymes relates to that of the common structure. Real ribozymes (similarly to SSCs measured above) have rather low degree of neutrality (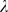) because these molecules have been produced by artificial directional selection [117], [118]. Such a decrease in robustness was shown. However, only 48.2% of the considered 305 real sequences (ribozymes and aptamers) have lower 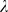 than the median for the random sequences. And 9.1% of the real sequences fall into the upmost decile, i.e. they have a higher 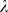 than 90% of random sequences; and 2.2% of the real sequences have higher 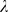 than 95% of the random sequences. All in all the distribution of neutralities is not different from the distribution obtained for the random sequences (see Methods). This is remarkable considering the fact that these ribozymes had been subject to intense directional selection for the required functionality. Although robustness and evolvability are not necessarily in conflict [119], [120], it is legitimate to ask whether stabilizing selection could increase the robustness of these populations further, as demonstrated in the theory of neutral networks [120]. We have thus exerted stabilizing selection on different molecules that already had a rather high 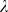 with population size 500 through 5000 generations (the only constraint was to maintain the phenotype). We show the highest degree of neutrality (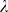) for structure-preserving variants (Fig. 5). It is apparent that stabilizing selection can guide robustness to the top 25% or even 5% of the distribution obtained for random sequences. Thus, we can expect that ribozymes in primordial ribo-organisms were even more error-resistant than ribozymes evolved *in vitro*, as they were subject to many generations of stabilizing selection.

## Discussion

We have found that the number of 1-step neutral mutants, for short sequences, is an excellent predictor of the error threshold (Fig. 2). Other characteristics of structure (see for example in [121]) are not as highly correlated with the error threshold. Maintenance of RNA secondary structure is a good predictor of maintenance of enzymatic activity [122], but especially around the active site the actual nucleotides presents are also important. In this investigation we have not considered critical sites in our fitness landscape, which would lower the degree of neutrality of sequences. Considering critical sites would most probably not affect the correlation of error threshold with the degree of neutrality.

The possibility of estimating the error threshold by available and easily computable characteristic of RNA sequences allows us to assess the replicability of aptamers and ribozymes. We have shown that functional phenotypes are mutationally robust above chance level and that, in effect, most known ribozymes could be replicated by a replicase working at the accuracy of the currently best RNA-dependent RNA polymerase ribozyme [17] (Fig. 4). Stabilizing selection, after the acquisition of function, can guide these molecular replicators to regions of sequence space which further increase robustness (Fig. 5).

It is important to discuss how our approach relates to the approach of Takeuchi *et al.* [15]. Their formula Eq. (2) was derived from heuristic considerations. We have explicitly numerically computed the error threshold for lengths up to 16 using the criterion of master phenoype to all others being 1∶1 in equilibrium concentrations. Note that since we calculate explicitly, back mutations naturally are accounted for and are thus are not neglected. One of the results of the present paper is that the “top-down” formula of Takeuchi *et al.* is in qualitative agreement with our bottom-up quantitative results. The relation between their critical parameter and ours is 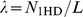. Using our 50% criterion for the error threshold we obtain the modified form of the Takeuchi-Hogeweg error threshold (Eq. (2)):
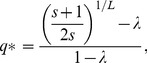
(4)


which does *not* agree quantitatively with our data (Fig. 3). This is why we have introduced the correction factor *α* accounting for back mutations in Eq. (3) under the assumption that back mutations from multiple deleterious mutants can be ignored. Note that the formula in Eq. (3) is non-linear but gives good fit for short sequences. With longer sequences the linear relationship between the error threshold and 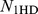 slowly deteriorates, and as shown in Fig. 3, there is increasing scatter around the nonlinear curve as well.

It is good news that *individually* all known ribozymes (genes) could be replicated in a realistic RNA world, but we must return to the important question as to how small genomes could have come into being. If we adopt the view that unlinked, naked genes preceded protocells and chromosomes [123] we should be happy with the current finding. There are mechanisms of dynamical coexistence of naked, unlinked replicators spreading on surfaces [124], [125]. In such a case, each sequence is competing with its own mutated copies (mutants can occasionally evolve into something new and useful [126]). We concur that such surface-bound dynamics was a stepping stone to “serious” forms of compartmentation, such as protocells [127]. Protocells can harbour a fair number of different, competing genes [128], but only if the error rate is low enough. It is plausible that error rates did evolve during the pre-cellular era of surface dynamics: more efficient (more accurate and faster) model replicases have been shown to spread on surfaces by kin selection [129]. We confirm the previous result in [19] that the transition from surface to protocell dynamics required only an order of magnitude increase in replication accuracy!

## Methods

### Derivation and analytical computation of the error threshold

The computation of the error threshold is based on the original quasispecies model of Eigen [1], [23], [24]:
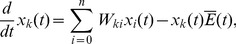
(5)


where *x_k_*(*t*) is density of sequence *k* at time *t*; the coefficients 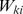 are elements of a value matrix **W** which contains replication and degradation rate constants (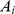
and 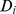, respectively) and mutation frequencies (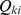) (the value matrix is filled according to the fitness landscape employed (see Results)):
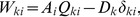
(6)


and 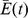
is the mean excess production:
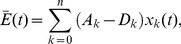
(7)


which can be removed by a non-linear transformation [23], [130]–[132] resulting an essentially linear equation. The model assumes that the only source of sequences is the correct or erroneous copies of present sequences; the substrates for replication are always present in sufficient quantity and excess molecules are washed out by a flux that keeps the total concentration constant.

As independent point mutations are assumed, mutation probability depends only on the Hamming distance of the initial (*i*) and final (*k*) binary sequences of length 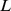:
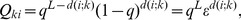
(8)


where 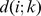 stands for the Hamming distance between the two sequences, 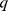 is the (constant) per digit replication accuracy, 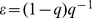.

The dynamics of the system is governed by the leading eigenvalue and the corresponding eigenvector of *W*. We assume that there is no degradation (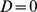), which does not affect the eigenvectors. Let 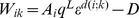 define the value matrix of the system and thus the modified equation without degradation is 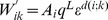. 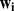 and 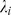 are eigenvectors and eigenvalues of the original matrix:
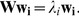
(9)


Consequently:



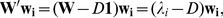
(10)


thus 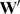 has the same eigenvectors and this type of transformation does not affect the rank of the eigenvalues.

The analytical solution of the system is the following, see e.g. [24], [132]:
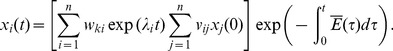
(11)


We are interested in the 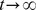 limit only. In this case:
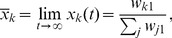
(12)


where 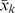 is the equilibrium mutant distribution, the “quasispecies” which consists of mutants distributed around the most efficient variant, called the master sequence.

Equation (3) can be solved either by integrating this ordinary differential equation numerically e.g. via Runge–Kutta method or computing the leading eigenvalue λ_1_ and the corresponding right eigenvector (**w**). We use the latter, simpler method because of its smoother behavior.

To compute the leading eigenvalue and the corresponding right eigenvector we used the Krylov-Schur method implemented in the *SLEPc* library [133] using the *PETSc* matrix routines [134].

We have computed the error threshold of a system in the following way: (1) The value matrix of Eq. (5) was filled according to the fitness landscape (high fitness 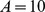, base fitness 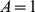), using 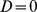. (2) The leading eigenvector **w** was computed at two per-digit replication accuracy values: 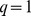 and 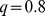 (in our systems, the error threshold always lies in this interval). From **w** it is easy to compute the total density of the master and mutant sequences. (3) The value of *q* at which the densities of the master and mutant sequences are the same is the error threshold (by our definition), thus we applied a secant algorithm to find the intersection point of the densities as a function of *q* (at a relative precision of 10^−6^). (4) The resulting 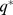 is the error threshold. The computation of an error threshold (with an arbitrary fitness landscape) using this algorithm – with a slight modification of the *SLEPc* code to reduce memory consumption – took about 12 hours and needed 4 GB of RAM on a 2.6 GHz Intel Xeon CPU.

### Analytical formulation of the phenotypic error threshold

Using the phenotypic dynamics described by Takeuchi *et al.* [15], the starting point is the following pair of differential equations:
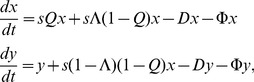
(13)


where *x* and *y* denote the focal phenotype and mutants, respectively; *Q* is the replication accuracy of *x*; Λ is the fraction of neutral mutants of 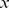; *D* is the constant degradation rate; 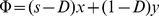 is the excess production; and *s* is the replication rate of the focal phenotype, while the mutants' replication rates are normalized to 1. We keep the concentration constant, i.e. 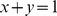. Computing the steady state solution for 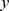 yields:
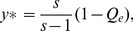
(14)


where
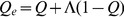
(15)


is the effective replication accuracy. Assuming that the number of neutral substitutions follows the binomial distribution (*q* denotes the correct per-digit replication probability):
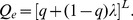
(16)


Our criterion for error threshold implies y* = 1/2. Combining these results, the critical per-digit replication accuracy (our error threshold) is
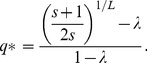
(17)


This calculation ignores back mutations. With the simple assumption that the frequency of back mutations is proportional (by a factor of α) to the number of 1-step mutants (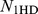), we get the following corrected critical per-digit replication accuracy:
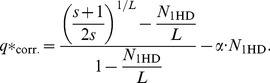
(18)


### Structural diversity of GC and GCAU sequences

Due to technical limitations, we have calculated the error threshold using sequences composed only of G and C, i.e. only two letters from the canonical four letter alphabet. Our question was: Would structural diversity, measured as the number of distinct structures and their relative frequencies, differ substantially for two and four letters?

**Figure 6:**
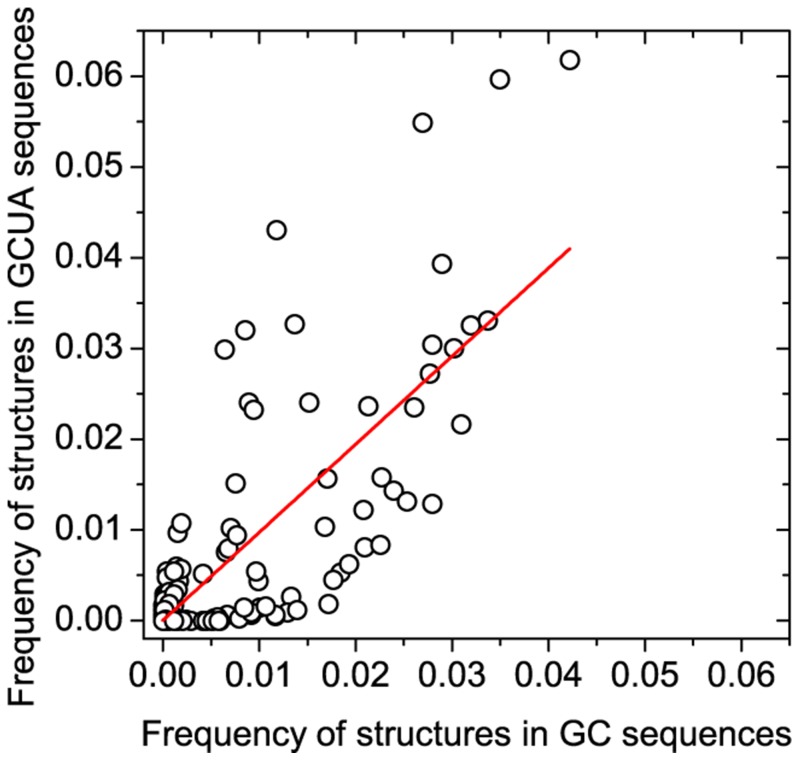
Correlation of structure frequencies of GC (two-letter) and GCAU (four-letter) sequences of length 14.

We have enumerated all sequences of length 14 using only GC nucleotides and using all four (GCAU). There are 16,384 unique GC sequences folding into 107 distinct structures (Table S2), whereas there are 268,435,456 unique GCAU sequences folding into 230 distinct structures (Table S2). In the case of the four-letter sequences, the most common structure (72.2%) is the one without any internal base-pair, in the binary sequences this structure has a much lower frequency (4.3%) due to the higher probability of having possible base-pairings in the sequence. If we leave out this structure, the relative frequencies of the remaining structures correlate in the two-base and four-base sequences. Correlation between frequencies is relatively high (0.79) (Fig. 6). A detailed investigation of RNA sequences of various alphabets can be found in [135].

### Calculation of the fraction of neutral 1-step mutants

For each sequence, the minimum free-energy structures of all 1-step mutants (differing only in one position from the original) are obtained. The number of 1-step mutants having the same structure as the original sequence is divided by the number of all possible 1-step mutants (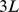) is the fraction of neutral one-mutants, 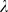.

Populations consisting of 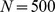 ribozymes were allowed to evolve for 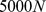 replications. At each replication each nucleotide of a sequence has a 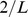 chance to mutate. This error rate is below the error threshold for all considered sequences. Sequences are chosen randomly for replication, with probability proportional to their structural similarity compared to the wild-type sequence. Thus we apply stabilizing selection on the structure of the wild-type sequence. After the 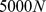 ^th^ replication, 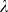 is calculated for each sequence folding to the original structure. The highest value among these is recorded.

### Statistical analysis

In order to statistically assess how the number of 1HD mutants of real ribozymes and aptamers before and after stabilizing selection relate to the distribution of number of 1HD mutants of random sequences, we calculated the percentile rank of data points in the random ensemble. Rank here means the average percentage ranking of the number of 1HD mutants. In case of multiple matches, average the percentage rankings of all matching scores. Percentiles are then divided into 10 bins of equal size between 0% and 100%. If the bins are equally populated then the distribution of the number of 1HD mutants for the real data is not different from that obtained for random sequences. Similarity of the distribution is assesses by 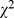 test. Multiple sequences come from the same study in our dataset, and thus the independency of the data point does not hold. Thus, we only use the sequences that are used in our analysis for further evolution, as we have only picked one from each study of similar length sequences. The distribution of the number of 1 HD mutants is not different from the distribution for random sequences (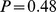). (Please note that for the whole set it would be 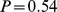, so the same.) After stabilizing selection is applied the distribution is markedly different from the one obtained for random sequences (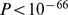).

## Supporting Information

Table S1
**Error threshold of real ribozymes and aptamers.** RNA sequences of ribozymes and aptamers from the literature is listed alongside their length, number of 1-neighbour neutral mutants, frequency of nearest-neighbour (one-step) neutral mutants (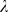), the estimated error threshold and the citation for the sequence. Stabilizing selection was applied to selected sequences, and the highest number of 1-neighbour neutral mutants is reported here.(XLSX)

Table S2 **Structural diversity of RNA sequences of length 14 with two letter (GC) and four letter (GCAU) alphabet.** Secondary structures in bracket notation are reported with the number of unique sequences folding to this structure. Frequencies of structures among all possible structures of length 14 are reported. The unstructured structure has the highest frequency among the sequences built from four letters. We also report the frequencies of structures if we omit these sequences from the total count.
(XLSX)

Table S3 **Secondary structure classes and Super secondary structure classes of GC sequences of length 16.** Secondary structures in bracket notation are reported with the number of unique sequences folding to this structure. The first column show the super secondary structure class (SSSC) without leading and trailing single stranded nucleotides. The second column gives the total number of unique sequences folding into the SSSC. Then in column 3 the individual structures are reported as well as their total unique sequence count (column 4).
(XLSX)
